# Characterization of quantum and classical correlations in the Earth’s curved space-time

**DOI:** 10.1038/s41598-020-71802-4

**Published:** 2020-09-07

**Authors:** Tonghua Liu, Shuo Cao, Shumin Wu

**Affiliations:** 1 Department of Astronomy, Beijing Normal University, Beijing, 100875 China; 2 Department of Physics, and Collaborative Innovation Center for Quantum Effects and Applications, Hunan Normal University, Changsha, 410081 Hunan China

**Keywords:** Physics, Information theory and computation, Quantum physics

## Abstract

The preparation of quantum systems and the execution of quantum information tasks between distant users are always affected by gravitational and relativistic effects. In this work, we quantitatively analyze how the curved space-time background of the Earth affects the classical and quantum correlations between photon pairs that are initially prepared in a two-mode squeezed state. More specifically, considering the rotation of the Earth, the space-time around the Earth is described by the Kerr metric. Our results show that these state correlations, which initially increase for a specific range of satellite’s orbital altitude, will gradually approach a finite value with increasing height of satellite’s orbit (when the special relativistic effects become relevant). More importantly, our analysis demonstrates that the changes of correlations generated by the total gravitational frequency shift could reach the level of $<0.5\%$ within the satellite’s height at geostationary Earth orbits.

## Introduction

As one of the most important developments in modern physics, quantum entanglement – a central characteristics of quantum correlations – has arouse widespread attention in most recent years^1,2^. However, one interesting question which still calls for consideration is, can the separable state (i.e., not entangled) be determined as a classically correlated state without quantum correlations? Several recent studies have focused on such issue and revealed some signatures of quantumness in separable states, one of which is the so-called quantum discord that captures the general quantum correlations even in the absence of entanglement in a quantum state. Following the methodology firstly proposed in^3–5^ and furthermore identified as a resource for computation^6^, quantum discord originates from the discrepancy between two classically equivalent definitions of mutual information, which can be derived from the measurement of the total correlations in a quantum state. Note that although the nature of quantum discord is still unknown, it is rewarding to investigate its important role played in the realization of some quantum information tasks (especially in the absence of quantum entanglement). One should remember that certain quantum information processing tasks can also be done efficiently, even without the participation of quantum entanglement^7,8^.

In realistic situation, the preparation of quantum system and the procession of quantum information tasks are always accompanied by gravitational and relativistic effects. Considering the fact that the previous works have paid more attention to quantum discord without gravitational or relativistic effects, its behaviors in a relativistic setting or curved space-time background is still an uncharted territory. Fortunately, the quantum field theory in curved space-time, which enables one to incorporate relativistic effects into quantum experiments, has provided a theoretical framework to carry out the above analysis^9,10^. Nevertheless, when studying quantum resource in relativistic setting, the effects of gravity and motion on the quantum properties and their applications have always been ignored, which fails to overcome the inherent inconsistency between quantum physics and relativity. Such gap was bridged by employing quantum field theory in curved space-time to compute the ultimate bounds on ultra-precise measurements of relativistic parameters^9,10^. More recently, further progress in this direction has been achieved by^11,12^, with two papers discussing quantum discord between relatively accelerated observers in de Sitter space. Meanwhile, it is of practical and fundamental importance to study the influence of gravitational effects on the quantum resources, especially when the parties involved are of distances apart in the curved space-time^13^. For instance, it was found in^14^ that there would be inevitable losses of quantum resources in the estimation of the Schwarzschild radius. Furthermore, a quantitative investigation of the dynamics of satellite-based quantum steering and coherence has been presented in^15,16^, given the curved background space-time of the Earth. Nowadays, it is possible to explore those quantum correlations with quantum discord acting as a resource in quantum protocols, as well as the correlations related to the practical implementation of many quantum information schemes in relativistic quantum systems, such as, quantum key distribution through satellite nodes^17^ and other quantum information tasks^18–23^.

In this work, we will quantitatively analyze how the curved space-time of the Earth influence the classical and quantum correlations (i.e., the losses of correlations), and furthermore discuss their behaviors under the Earth’s gravitational effects. The correlated photon pairs are initially prepared in a two-mode squeezed state, one of which is located on the Earth’s surface and the other is propagating to a satellite. Note that in the propagating process, the photons’ wave-packet will be deformed by the curved background space-time and a lossy quantum channel can be used to model these deformed effects on the quantum state of photons^24^. The advantage of this work is that the Earth’s gravitational field is described by a lossy channel rather than global free models, because the latter suffer from the single-mode approximate problem and physically unfeasible detection in the full space-time^25^. Meanwhile, our results could be in principle applied to all types of correlations affected by the acceleration physical system, according to the equivalence principle in which the effects of gravity are exactly equivalent to those of acceleration.

This paper is organized as follows. Firstly, we describe the quantum field theory of a massless uncharged bosonic field propagating from the Earth to a satellite. Secondly, we briefly introduce the definition of the measurements of mutual information, classical correlation, and quantum correlation (or the quantum discord) for a bipartite Gaussian state. Thirdly, we show a scheme to test long-distance quantum discord, based on which the behaviors of three types of correlations will be studied in the curved space-time. Throughout the paper we employ natural units of $G = c =\hbar = 1$.

## Results

### Light wave-packets propagating in the Earth’s space-time

In this section, we will give a brief introduction to the propagation of photons under the influence of the Earth’s gravitational field. Considering the rotation of the Earth, the space-time considered in this analysis can be approximately described by the Kerr metric^26^ and our work will be performed on the equatorial plane for simplicity. Now the Kerr line element in the Boyer-Lindquist coordinate $(t,r,\phi )$ is reduced to^26^

1 $$\begin{aligned} ds^2&= -\,\Big (1-\frac{2M}{r} \Big )dt^2+\frac{1}{\Delta }dr^2 \nonumber \\&\quad +\Big (r^2+a^2+\frac{2Ma^2}{r}\Big ) d\phi ^2 - \frac{4Ma}{r} dt \, d\phi , \end{aligned}$$

2 $$\begin{aligned} \Delta&=1-\frac{2M}{r}+\frac{a^2}{r^2}, \end{aligned}$$

where *M* and *r* respectively denote the mass and radius of the rotating planet. The Kerr parameter (i.e., normalized angular momentum) can be expressed as $a=\frac{J}{M}$, a combination of the planet’s angular momentum (*J*) and mass *M*.

In order to clearly describe the propagation of wave-packets from a source on Earth to a receiver satellite at a fixed distance, two observers called Alice and Bob are respectively prepared as the reference frames. More specifically, focusing on a photon sent from Alice at the time of $\tau _A$, it will arrive at Bob at the time of $\tau _B=\Delta \tau +\sqrt{f(r_B)/f(r_A)}\tau _A$, where *f*(*r*) is the gravitational frequency shifting factor and the $\Delta \tau$ represents the propagation time of the light from Alice to Bob. As is well known, photons can be modeled by the wave packet of massless bosonic field with a distribution of $F^{(K)}_{\Omega _{K,0}}$, where $\Omega _{K}$ is the mode frequency peaked at $\Omega _{K,0}$^27,28^ and $K=A, B$ denotes the mode in Alice’s or Bob’s reference frame. For an observer infinitely far away from Alice or Bob, the annihilation operator takes the form of

3 $$\begin{aligned} \hat{a}_{\Omega _{K,0}}(t_K)=\int _0^{+\infty }d\Omega _K e^{-i\Omega _K t_K}F^{(K)}_{\Omega _{K,0}}(\Omega _K)\hat{a}_{\Omega _K}, \end{aligned}$$

with the frequency distribution of $F^{(K)}(\Omega )$. Such operator is naturally applied to modeling a wave packet of the electromagnetic field located and propagating in the space-time. Note that when the frequency distribution $F^{(K)}(\Omega )$ is normalized (i.e., $\int _{\Omega >0}|F^{(K)}(\Omega )|^2=1$), the creation $\hat{a}^{\dagger }_{\Omega _{K,0}}$ and annihilation $\hat{a}_{\Omega _{K,0}}$ operators will satisfy the canonical equal time bosonic commutation relations, ($[\hat{a}_{\Omega _{K,0}}(t),\hat{a}^{\dagger }_{\Omega _{K,0}}(t)]=1$).

**Figure 1:**
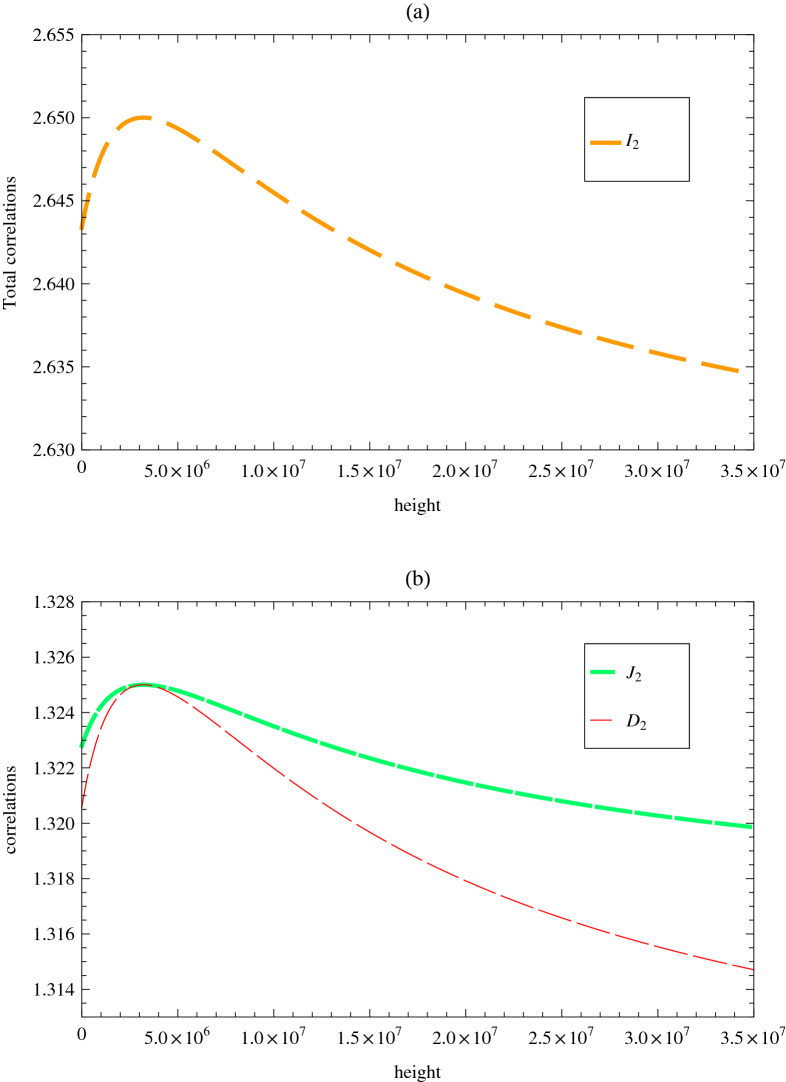
Three types of correlations (${I}_2$, ${J}_2$ and ${D}_2$) as functions of increasing orbit height *h*. The Gaussian bandwidth, squeezing parameter and frequency of mode $b_2$ are fixed at $\sigma =1$, $s=1$ and $\Omega _B=1$, respectively.

**Figure 2:**
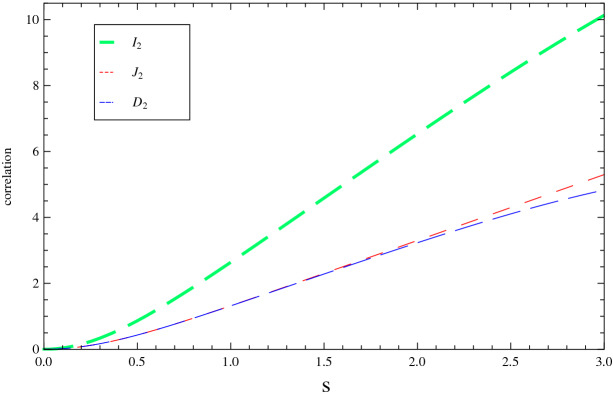
Three types of correlations (${I}_2$, ${J}_2$ and ${D}_2$) as functions of the squeezing parameter *s*. The orbit height of the satellite, frequency of mode $b_2$ and the Gaussian bandwidth are fixed at $h=2\times 10^4$ km, $\Omega _B=1$ and $\sigma =1$, respectively.

Now let us consider a realistic case in which Alice (located on the surface of the Earth, $r_A=r_E$) prepares and sends a wave packet $F^{(A)}_{\Omega _{A,0}}$ to Bob (located on a satellite at the altitude of $r_B$). Given the effects of the Earth’s gravitational field, the wave packet received by Bob ($F^{(B)}_{\Omega _{B,0}}$) should be modified, following the relation between the frequency distributions of two wave packets^9,10^. Meanwhile, the gravity of the Earth also changes the mode frequency $\Omega _K$ with the shift of $\Omega _A=\sqrt{f(r_A)/f(r_B)}\Omega _B$, where $f(r_A)/f(r_B)$ represents the shifting function of total gravitational frequency (see more details in the text). Therefore, the total modification induced by the Earth’s gravitational field can be parameterized as

4 $$\begin{aligned} F^{(B)}_{\Omega _{B,0}}(\Omega _B)=\root 4 \of {\frac{f(r_B)}{f(r_A)}}F^{(A)}_{\Omega _{A,0}}\left( \sqrt{\frac{f(r_B)}{f(r_A)}}\Omega _B\right) . \end{aligned}$$

One may clearly see that the effect induced by the curved space-time of the Earth cannot be simply corrected by a linear shift of frequency, which indicates the difficulty of compensating such transformation in realistic implementations.

Fortunately, following the relation between such nonlinear gravitational effect and the fidelity of the quantum channel^9,10^, it is possible to decompose the mode $\bar{a}^{\prime }$ received by Bob into

5 $$\begin{aligned} \bar{a}^{\prime }=\Theta \hat{a}^{\prime }+\sqrt{1-\Theta ^2}\hat{a}_{\bot }^{\prime }, \end{aligned}$$

in terms of the mode prepared by Alice ($a^{\prime }$) and its orthogonal mode $\hat{a}_{\bot }^{\prime }$ (i.e., $[\hat{a}^{\prime },\hat{a}_{\bot }^{\prime \dagger }]=0$)^29^. Here $\Theta$ is the wave packet overlap between the distributions $F^{(B)}_{\Omega _{B,0}}(\Omega _B)$ and $F^{(A)}_{\Omega _{A,0}}(\Omega _B)$, which takes the form of

6 $$\begin{aligned} \Theta :=\int _0^{+\infty }d\Omega _B\,F^{(B)\star }_{\Omega _{B,0}}(\Omega _B)F^{(A)}_{\Omega _{A,0}}(\Omega _B). \end{aligned}$$

It is easy to see that $\Theta =1$ corresponds to a perfect channel, while $\Theta <1$ represents a noisy channel under the influence of the Earth’s curved space-time. In order to better characterize the frequency distribution of the source, we introduce a specific quantity, i.e., fidelity of ${F}=|\Theta |^2$ in the following analysis and apply a real normalized Gaussian wave packet to Alice’s mode

7 $$\begin{aligned} F_{\Omega _0}(\Omega )=\frac{1}{\root 4 \of {2\pi \sigma ^2}}e^{-\frac{(\Omega -\Omega _0)^2}{4\sigma ^2}}, \end{aligned}$$

with the wave packet width of $\sigma$. We remark here that in the expression of the overlap parameter $\Theta$ (Eq. (6)), the integration will be performed over strictly positive frequencies, which is justified by the fact that the peak frequency is typically much larger than the spreading of the wave packet (i.e.,$\Omega _0\gg \sigma$). The combination of Eqs. (4) and (7) provides us with

8 $$\begin{aligned} \Theta =\sqrt{\frac{2}{1+(1+\delta )^2}}\frac{1}{1+\delta }e^{-\frac{\delta ^2\Omega _{B,0}^2}{4(1+(1+\delta )^2)\sigma ^2}}, \end{aligned}$$

where the new parameter $\delta$ is introduced to quantify the shifting effect

9 $$\begin{aligned} \delta =\root 4 \of {\frac{f(r_A)}{f(r_B)}}-1=\sqrt{\frac{\Omega _B}{\Omega _A}}-1. \end{aligned}$$

Focusing on the equatorial plane of the Earth described by Kerr metric, the parameter of $\frac{\Omega _B}{\Omega _A}$ may be rewritten as^30^

10 $$\begin{aligned} \frac{\Omega _B}{\Omega _A}=\frac{1+\epsilon \frac{a}{r_B}\sqrt{\frac{M}{r_B}}}{C\sqrt{1-3\frac{M}{r_B}+ 2\epsilon \frac{a}{r_B}\sqrt{\frac{M}{r_B}}}}. \end{aligned}$$

Here the normalization constant takes the form of $C=[1-\frac{2M}{r_A}(1+2a {\omega })+\big (r^2_A+a^2-\frac{2Ma^2}{r_A}\big ){\omega }^2]^{-\frac{1}{2}}$ (with the Earth’s equatorial angular velocity $\omega$) and $\epsilon =\pm 1$ stands for the direction of the corresponding orbit (i.e., the satellite co-rotates with the Earth when $\epsilon =+1$).

Now we will expand Eq. (9) by keeping the first order of the perturbation term $(r_A \omega )^2$, in order to derive the explicit expression of the frequency shift ($\delta$) for a photon exchanged between Alice and Bob. Considering the independency between the perturbative result and the state of the Earth and the satellite (i.e., whether they are co-rotating with each other), one can easily obtain the shift parameter with the following expression

$$\begin{aligned} \delta&= \delta _{Sch}+\delta _{rot}+\delta _h\\&= \frac{1}{4}\frac{r_S}{r_A}\big (\frac{r_A-2h}{r_A+h} \big )-\frac{(r_A\omega )^2}{2}-\frac{(r_A\omega )^2}{4}\big (\frac{3}{4}\frac{r_S}{r_A}-\frac{2r_Sa}{\omega r_A^3}\big ), \end{aligned}$$

where $\delta _{Sch}$, $\delta _{rot}$, and $\delta _h$ respectively denote the first-order Schwarzschild term, rotation term, and higher-order correction term. Note that $r_S=2M$ is the Schwarzschild radius of the Earth, while the parameter $h=r_B-r_A$ quantifies the height difference between Bob and Alice. It should be pointed out that when $h=\frac{r_A}{2}$, the overlap parameter $\Theta$ is no longer equal to one due to the effects induced by the rotation of the Earth. However, when the satellite moves at the height of $h\simeq \frac{r_A}{2}$, the combined effects of the Earth’s gravity and Special Relativity (i.e., the Doppler effect generated by the motion of the satellite) will compensate each other ($\Theta =1$). Therefore, the photon received by Bob at this height will not experience any frequency shift, which implies that the clock rate of Bob will become equal to that of Alice.

### The influence of Earth’s curved space-time on three types of correlations

In this section, we propose a scheme to test the classical and quantum correlations at long distance, and furthermore quantify the effects of the Earth’s curved space-time on such correlations. In the framework of a pair of entangled photons initially prepared in a two-mode squeezed state (with the modes of $b_1$ and $b_2$ on the Earth’s surface), we firstly send a photon (with the mode $b_1$) to Alice, with the other photon (with the mode $b_2$) propagating from the Earth to the satellite and then received by Bob. Now the wave packet of photons will be deformed by the curved background space-time of the Earth, based on which the total correlation, classical correlation, and quantum correlation (quantum discord) of this system will be investigated in the curved space-time.

Considering the fact that the two modes ($b_1$ and $b_2$) will be received by Alice and Bob at different heights (i.e., within different satellite orbits), the effects caused by the curved space-time should be taken into account in the total process. As was extensively discussed in^9,10^, such space-time effects on the two-mode squeezed state can be modeled by a beam splitter with the orthogonal modes of $b_{1\bot }$ and $b_{2\bot }$. The covariance matrix of the initial two-mode squeezed state is given by

11 $$\begin{aligned} \Sigma ^{b_1b_2b_{1\bot }b_{2\bot }}_0=\left( \begin{array}{cc} \tilde{\sigma }(s) & 0 \\ 0 & I_4 \end{array}\right) , \end{aligned}$$

where ${I}_4$ represents the $4\times 4$ identity matrix and $\tilde{\sigma }{(s)}$ denotes the covariance matrix of the two-mode squeezed state

12 $$\begin{aligned} \tilde{\sigma }(s)=\left( \begin{array}{cc} \cosh {(2s)} {I}_2&\sinh {(2s)}\sigma _z \\ \sinh (2s)\sigma _z &\cosh {(2s)} {I} _2 \end{array}\right) , \end{aligned}$$

with the Pauli matrix $\sigma _z$ and squeezing parameter *s*. Following the recent analysis of^9,10^, one can use lossy channels to model such effects and we only consider Bob’s mode sent to the satellite and Alice’s mode sent to the ground, which means that Alice will experience a perfect channel ($\Theta _1=1$). Therefore, in our scheme one may naturally obtain

13 $$\begin{aligned} \bar{b}_1 &= \,b_1\nonumber \\ \bar{b}_2& = \Theta _2\,b_2+\sqrt{{1-\Theta _2^2}}b_{2\bot }, \end{aligned}$$

and such process can be described by a mixed beam splitting of two modes, $b_1(b_2)$ and $b_{1\bot }(b_{2\bot })$. For the entire process, the symplectic transformation may be encoded into the Bogoloiubov transformation

$$\begin{aligned} S=\left( \begin{array}{cccc} {I}_2 &0& 0&0 \\ 0&\Theta _2 {I} _2 &0&\sqrt{ 1-\Theta _2^2} {I} _2\\ 0 &0& -{I}_2&0 \\ 0&\sqrt{ 1-\Theta _2^2} {I} _2 &0&-\Theta _2 {I} _2 \end{array}\right) , \end{aligned}$$

based on which the final state after transformation is defined as $\Sigma ^{b_1b_2b_{1\bot }b_{2\bot }}=S\,\Sigma _0^{b_1b_2b_{1\bot }b_{2\bot }}\,S^{T}$. Then one could trace over the orthogonal modes ($b_{1\bot },b_{2\bot }$) and obtain the covariance matrix $\Sigma ^{b_1b_2}$ for the modes ($b_1$ and $b_2$) after propagation

14 $$\begin{aligned} \Sigma ^{b_1b_2}=\left( \begin{array}{cc} (1+2\sinh ^2s ) {I}_2 &\sinh {(2s)}\,\Theta _2\,\sigma _z \\ \sinh {(2s)}\,\Theta _2\,\sigma _z &(1+2\sinh ^2s\,\Theta _2^2 )\, {I}_2 \end{array}\right) . \end{aligned}$$

In our analysis, Eq. (14) will be used to characterize the final form of the two-mode squeezed state that suffers from the influence of the Earth’s gravity.

**Figure 3:**
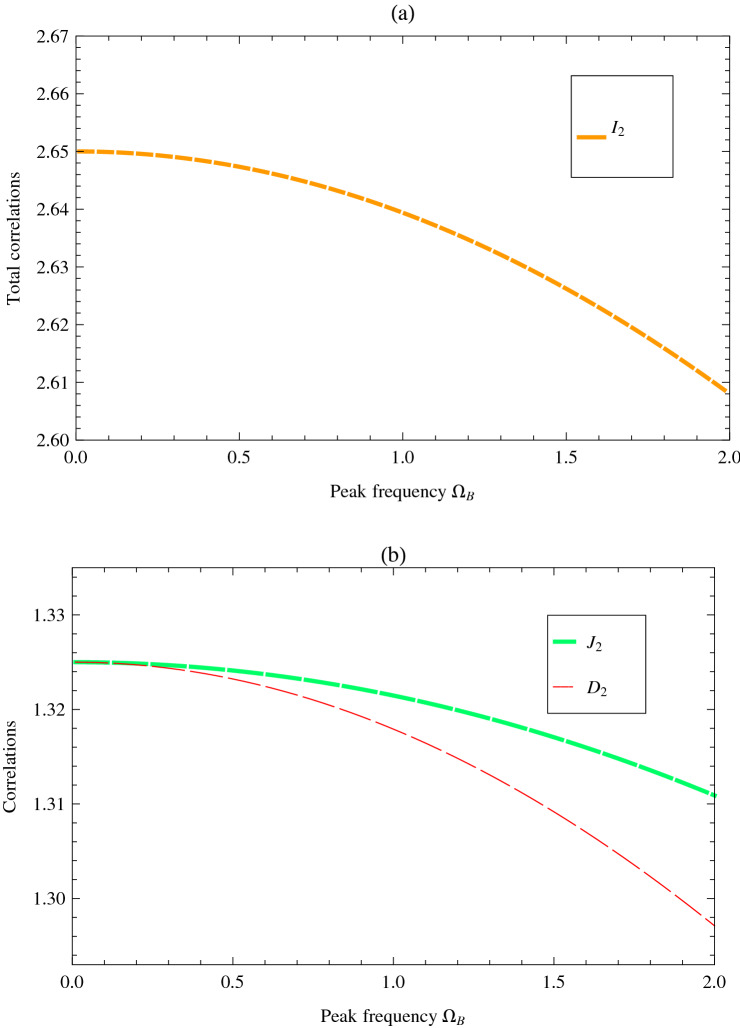
Three types of correlations (${I}_2$, ${J}_2$ and ${D}_2$) in terms of the peak frequency of mode $b_2$. The other relevant parameters are fixed at $s=1$, $\sigma =1$ and $h=2\times 10^4$ km.

**Figure 4:**
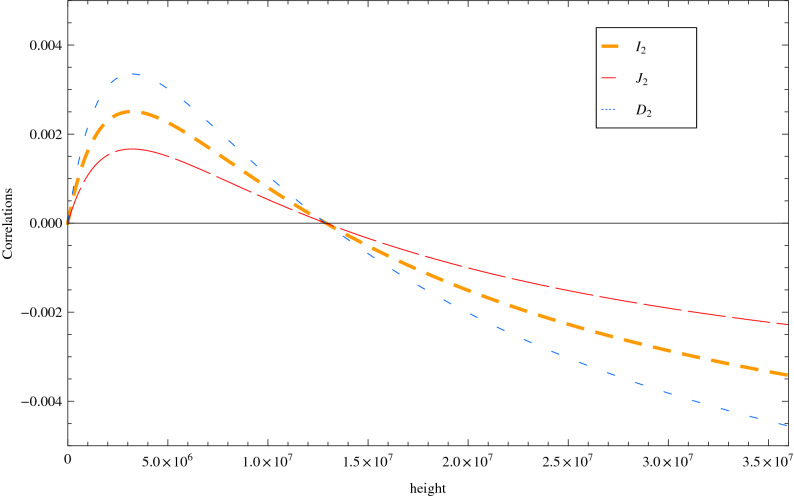
The change rate of three types of correlation $\mu _C$ in term of the satellite’s orbital height. The other state parameters are fixed at $s=1$, $\sigma =1$ and $\Omega _B=1$.

Let’s remark here that a lossy quantum channel determined by the wave packet overlap parameter $\Theta$ (which contains the parameters of $\delta$, $\sigma$ and $\Omega _{B,0}$), could fully describe the effect of the Earth’s curved space-time on the quantum state. For a typical case that satellites stay within the geosynchronous satellite orbit, one could straightforwardly obtain the shift parameter as $\delta \sim 2.5\times 10^{-10}$, based on the value of the Schwarzschild radius of the Earth ($r_S=9$ mm). In addition, we also focus on a typical parametric down converter crystal (PDC) source^42,43^ with a wavelength of 598 nm and Gaussian bandwidth of $\sigma =1$MHz (which corresponds to the peak frequency of $\Omega _{B,0}= 500$ THz). Given the condition of $\delta \ll (\frac{\Omega _{B,0}}{\sigma })^2\ll 1$, we can expand the wave packet overlap $\Theta$ by keeping the second-order term of the shift parameter, i.e., $\Theta \sim 1-\frac{\delta ^2\Omega _{B,0}^2}{8\sigma ^2}$. In order to ensure the validity of perturbation expansion for the Matrix element, we furthermore estimate the value of $\frac{\delta ^2\Omega ^2_{B,0}}{8\sigma ^2}\sim 3.2\times 10^{-8}$, based on which one may guarantee the validity of the perturbative expansion with the squeezing parameter $s\ll 7.6$ (which corresponds to $\sinh ^2(s)\ll 10^6$). For convenience, the peak frequency and the Gaussian bandwidth are respectively rescaled to

15 $$\begin{aligned} \tilde{\Omega }\equiv \frac{\Omega }{\Omega _{B,0}}, \tilde{\sigma }\equiv \frac{\sigma }{\sigma _0}, \end{aligned}$$

with $\Omega _{B,0}=500$ THz and $\sigma _0=1$ MHz. In the following analysis, the dimensionless parameters $\tilde{\Omega }$ and $\tilde{\sigma }$ will be abbreviated as $\Omega$ and $\sigma$, respectively.

Summarizing, the effects of the Earth’s curved space-time will quantified by three types of correlations, i.e., the total correlation ${I}_2$, classical correlation ${J}_2$ and quantum discord ${D}_2$ between the two modes of $b_1$ and $b_2$ (see the Methods for more details). The behaviors of ${I}_2$, ${J}_2$ and ${D}_2$ are explicitly shown in in Fig. 1, which illustrates the three types of correlations as functions of increasing orbit height *h*. The Gaussian bandwidth, squeezing parameter and frequency of mode $b_2$ are respectively fixed at $\sigma =1$, $s=1$ and $\Omega _B=1$. One may clearly see that, compared with the classical correlation, the quantum discord will be more easily effected by the Earth’s curved space-time. Such tendency has been firstly noted and extensively discussed in the previous works^44^. Moreover, the three type of correlations between mode $b_1$ and $b_2$ have exhibited very similar behaviors, i.e., they all initially increase for a specific range of height parameter $h\simeq \frac{r_A}{2}$ and then gradually approach to a finite value with increasing *h*. One possible explanation to such findings lies in the different roles played by gravitational frequency shift and special relativistic effects, with the increase of the satellite’s height. More specifically, the photon’s frequency received by the satellites at the height of $h<\frac{r_A}{2}$ will experience blueshift (with increasing correlations), while the corresponding frequencies received at the height of $h>\frac{r_A}{2}$ will experience redshift (with decreasing correlations). Actually, in the framework of three types of correlations between the photon pairs, the peak values of all correlations (i.e., the parameter $\delta =0$) have strongly suggested a detectable frequency transformation from blueshift to redshift^30^. It should be pointed out that the total frequency shift generated by both special and general relativistic effects should be taken into account (Eq. (9)), when the two parties are located at the same height or in the flat space-time ($\delta \ne 0$)^30^. In addition, when the satellite is moving at the height of $h=\frac{r_A}{2}$ with vanishing Schwarzschild term ($\delta _{Sch}$), photons received on the satellite will generate a tiny frequency shift, in the case of which the lowest-order rotation term $\delta _{rot}$ and higher-order correction term $\delta _{h}$ should be taken into consideration.

In order to better understand the relations between the three different correlations and the initial squeezing parameter, in Fig. 2 we also show the evolution of ${I}_2$, ${J}_2$ and ${D}_2$ with *s*, with fixed value for the orbit height at geostationary Earth orbits ($h=3.6\times 10^4$ km), the frequency of mode ($b_2=1$), and the Gaussian bandwidth ($\sigma =1$). It is apparent that although all of the three type of correlations will increase with the squeezing parameter, the mutual information ${I}_2$ is much more sensitive to the change of squeezing parameter *s*, compared with the classical correlation and the quantum discord. Similarly, considering the degeneracy between the wave packet overlap parameter $\Theta$ and the frequency parameter $\Omega _B$, it is also necessary to investigate the change of ${I}_2$, ${J}_2$ and ${D}_2$ with $\Omega _B$. The results are illustrated in Fig. 3. Besides the general tendency that all types of correlations will decrease with the frequency parameter, one could also find that the quantum discord ${D}_2$ is more sensitive to the change of frequency parameter $\Omega _B$. Such conclusion strongly implies the future possibility of choosing appropriate parameters to realize quantum communications from the Earth to the satellite.

Finally, with the aim of better quantifying the influence of the Earth’s curved space-time, we will define an additional quantity to describe the change rate of three types of correlations

16 $$\begin{aligned} \mu _C=\frac{C(\Sigma ^{b_1b_2})-C_0(\Sigma ^{b_1b_2})}{C_0(\Sigma ^{b_1b_2})}, \end{aligned}$$

where $C={I}_2, {J}_2,{D}_2$ and the subscript 0 denotes its corresponding value at the height of the satellite ($h=0$). In Fig. 4 we plot the change rate of correlation $\mu _C$ as a function of the height parameter *h*, with fixed Gaussian bandwidth $\sigma =1$ and frequency parameter $\Omega _B=1$. The behavior of the $\mu$ parameter in three types of correlations has clearly demonstrates the effects of blueshift and redshift at the height of $h<\frac{r_A}{2}$ and $h>\frac{r_A}{2}$. Therefore, our analysis has revealed a special height of $h=2r_A$ (which corresponds to $\delta =-\,1$) at which the blueshift and the redshift effects might cancel out with each other. More interestingly, we find that the changes of correlations generated by the total gravitational frequency shift could reach the level of $<0.5\%$ within the satellite’s height at geostationary Earth orbits.

## Discussion

In conclusion, we have studied the influence of the Earth’s curvature on three types of correlations including total correlation (mutual information), classical correlation, and quantum correlation (quantum discord) for a two-mode Gaussian state, in which one of the modes is propagating from the ground to a satellite. Different from the Schwarzschild case (with no rotation) widely discussed in the previous works, our analysis concentrates on a general case that the special relativistic effects are involved and the space-time around the Earth is described by the Kerr metric (considering the rotation of the Earth). Our results indicate that all of the three types of quantum correlations which initially increase for a specific range of satellite’s orbital altitude, will gradually approach a finite value with increasing height of satellite’s orbit (when the special relativistic effects become relevant). Meanwhile, our quantitative analysis suggests that although all of correlations will increase with the squeezing parameter, the mutual information is more sensitive to the squeezing parameter *s*, compared with the classical correlation and quantum discord. Focusing on the degeneracy between the correlations and frequency parameter, our analysis demonstrates the inverse relation between the frequency parameter $\Omega _B$ and three types of correlations, i.e., a lower peak frequency parameter will lead to less loss. However, one could also perceive that the quantum discord is more sensitive to the change of frequency parameter $\Omega _B$ than the classical correlation. Finally, in the framework of a quantity describing the change rate of three types of correlations, we detect a special height ($h=2r_A$) at which the blueshift and redshift effects are offset. More importantly, the changes of correlations generated by the total gravitational frequency shift is determined at the level of $<0.5\%$ within the satellite’s height at geostationary Earth orbits. Such findings could provide some interesting possibilities to reduce the loss of three types of correlations (especially the quantum discord) through the control of the satellite’s orbital height.

Therefore, with the rapid developments in quantum technology and quantum communication, it is quite possible to implement quantum tasks between the ground and satellites with the three types of correlations studied in this work. Our results could also contribute to the future study of multi-particle quantum states, which should be taken into account in the realization of quantum information and communication tasks in multi-particle systems. More specifically, when the initial state is taken as multi-particle quantum state, the final state after propagation also can be obtained by calculating the covariance matrix. As a final remark, considering the fact that realistic quantum systems always exhibit gravitational and relativistic features, our analysis in this paper can be extended to the investigation of the dynamics of all types of correlation under the influence of acceleration. Such conclusion is supported by the equivalence principle in General Relativity, which states that the effects of gravity are exactly equivalent to the effects of acceleration.

## Methods

In the framework of a general two-mode Gaussian state ($\rho _{AB}$) including two subsystems (*A* and *B*)^31^, the vector of the field quadratures $\hat{R} = (\hat{x}^A,\hat{p}^A, \hat{x}^B,\hat{p}^B)^\mathsf{T}$ satisfies the canonical commutation relations $[{{{\hat{R}}_k},{{\hat{R}}_l}} ] = i{\Omega _{kl}}$ with the symplectic form of $\Omega = {{\ 0\ \ 1}\atopwithdelims (){-1\ 0}}^{\bigoplus {2}}$. Note that all of the Gaussian properties can be determined from the symplectic form of the covariance matrix (CM) $\mathbf {\sigma }_{ij} = \text {Tr}\big [ {{{\{ {{{\hat{R}}_i},{{\hat{R}}_j}} \}}_ + }\ {\rho _{AB}}} \big ]$^32–35^, which is transformed into a standard form through the diagonal subblocks

17 $$\begin{aligned} \mathbf {{\sigma }}_{AB}= \left( \begin{array}{cc} A&C\\ C^T&B \end{array}\right) , \end{aligned}$$

with $A=a\mathbb {I}$, $B=b\mathbb {I}$, and $C=diag \{c_1,c_2\}$. The symplectic eigenvalues of $\sigma _{AB}$ are given by $2{\nu }_{\mp }^2=\Delta \mp \sqrt{\Delta ^2-4\det \sigma _{AB}}$, with $\Delta =\det A+\det B+2\det C$. See^34–38^ for more details about the structural and formal description of the Gaussian quantum states in the phase space. Now we will briefly introduce the definition of the mutual information (or total correlation), classical correlation, and quantum correlation (or quantum discord) in the continuous variable case^3–5^.

$\textit{Total correlation or mutual information.}$ The mutual information, or a measurement of the total correlations in a quantum state, is often used to describe the amount of information included in a quantum state. Instead of the von Neumann entropy commonly used in the previous studies, in this analysis we will focus on the Rényi entropy of order 2 to quantify all of the relevant quantities with clear and detailed expression, which satisfy the strong subadditivity inequality for arbitrary Gaussian states^39^. Now the total correlation between *A* and *B* can be quantified by the Rényi entropy mutual information

18 $$\begin{aligned} {I}_2(\sigma _{AB})&= {S}_2(\sigma _A)+{S}_2(\sigma _B)-{S}_2(\sigma _{AB})\nonumber \\ &= \frac{1}{2}\ln \big (\frac{\det A\det B}{\det \sigma _{AB}}\big )\ , \end{aligned}$$

with the Rényi entropy of ${S}_2(\sigma )=\frac{1}{2}\ln (\det \sigma )$ and a given Gaussian state of $\sigma _{AB}$.

$\textit{Classical correlation.}$ As is well known, the one-way classical correlation is always obtained by local measurements. Specially, when the most informative local measurement is performed on subsystem *B*, we can define ${J}_2(\sigma _{A|B})$ to quantify how much the ignorance about the state of subsystem, i.e., *A*, is reduced when the most informative local measurement is performed on subsystem *B*.

*A* is reduced. Based on the newly-introduced Gaussian Rényi-2 measurement of the one-way classical correlation, ${J}_2(\sigma _{A|B})$ could be interpreted as the maximum decrease in the Rényi-2 entropy of subsystem *A*, given a Gaussian measurement performed on subsystem *B*. Note that the maximization is realized by mapping Gaussian states to Gaussian state in the continuous variable system, which generates the expression of ${J}_2(\sigma _{A|B})$ as

19 $$\begin{aligned} {J}_2(\sigma _{A|B})& = {S}_2(\sigma _A)-\inf _{\Pi _B(\eta )}{S}_2(\tilde{\sigma }_{A})\nonumber \\& = \sup _{\Pi _B(\eta )} \frac{1}{2}\ln \big (\frac{\det A}{\det \tilde{\sigma }_{A}}\big )\ , \end{aligned}$$

Here $\Pi _B(\eta )$ denotes the Gaussian measurement on subsystem *B*. One could obtain CM $\tilde{\sigma }_{A}$ through the conditional state of subsystem *A* ($\rho _{A|\eta }$), after a measurement of $\Pi _B(\eta )$ performed on subsystem *B* with the outcome of $\eta$. It’s worth noting that the Gaussian measurements used in this analysis denote Gaussian Positive Operator Valued Measurements (POVMs), benefit from its extensive applications in linear optics and homodyne detection^40^. The expression of ${J}_2(\sigma _{B|A})$ could be straightforwardly obtained by swapping the roles of the two subsystems $A\leftrightarrow B$^41^.

$\textit{Quantum correlation or quantum discord.}$ The quantum correlation (or quantum discord) is originated from the discrepancy between two classically equivalent definitions of mutual information. As a measurement of the quantumness of correlations, its definition based on the Rényi-2 entropy can be expressed as

20 $$\begin{aligned} \nonumber {D}_2(\sigma _{A|B})&= {I}_2(\sigma _{AB})-{J}_2(\sigma _{A|B})\\&= \inf _{\Pi _B} \frac{1}{2}\ln \big (\frac{\det B\det \tilde{\sigma }_{A}}{\det \sigma _{AB}}\big )\ , \end{aligned}$$

Similarly, the expression of ${D}_2(\sigma _{B|A})$ could be straightforwardly obtained by swapping the roles of the two subsystems $A\leftrightarrow B$. As a final remark, one can clearly see that the quantum correlation could be explained as the difference between the total and classical correlation, based on the definitions of the three types of correlations shown above.
